# Structure and regulation of coronavirus genomes: state-of-the-art and novel insights from SARS-CoV-2 studies

**DOI:** 10.1042/BST20200670

**Published:** 2020-12-24

**Authors:** Ilaria Manfredonia, Danny Incarnato

**Affiliations:** Department of Molecular Genetics, Groningen Biomolecular Sciences and Biotechnology Institute (GBB), University of Groningen, Nijenborgh 7, 9747 AG, Groningen, The Netherlands

**Keywords:** coronavirus, RNA structure, SARS-CoV-2

## Abstract

Coronaviruses (CoV) are positive-sense single-stranded RNA viruses, harboring the largest viral RNA genomes known to date. Apart from the primary sequence encoding for all the viral proteins needed for the generation of new viral particles, certain regions of CoV genomes are known to fold into stable structures, controlling several aspects of CoV life cycle, from the regulation of the discontinuous transcription of subgenomic mRNAs, to the packaging of the genome into new virions. Here we review the current knowledge on CoV RNA structures, discussing it in light of the most recent discoveries made possible by analyses of the SARS-CoV-2 genome.

## Introduction

RNA viruses represent one of the most common classes of pathogens behind human diseases [1], with around 180 currently recognized species, and around three new species discovered every year [2]. The harmfulness of these viruses is partially supported by their ability to rapidly evolve and adapt, allowing easier escape of host immune responses and quicker development of resistance towards drugs and vaccines. This ability relies on the low fidelity of viral RNA polymerases. The lack of any proofreading activity results in mutation rates as high as 10^−3^ [3], nearly 6–7 orders of magnitude higher than those of bacterial DNA polymerases [4].

The RNA genomes of these viruses carry two layers of information [5]. On one layer, the primary sequence encodes for all the viral proteins needed to hijack the host cell machinery and to generate new viral particles.

The second layer of information relies on the ability of RNA to fold into stable secondary structures, by base-pairing between regions of internal complementarity, followed by further compaction into tertiary structures. RNA structure elements, as well as their ability to dynamically interconvert between alternative conformations, have been widely proven to be essential players in the life cycle of various RNA viruses. As an example, the HIV trans-activation response (TAR) element, that can switch between two alternative conformations, is essential for the Tat-mediated activation of viral replication, it provides the docking site for the interaction with several host proteins, and it can be further processed into a mature microRNA to repress target host mRNAs [6–9].

In this perspective, recent technological advances based on RNA structure probing approaches coupled to next-generation sequencing (NGS) technologies [5,10,11] are progressively expanding our ability to query higher-order structures in the genome of RNA viruses, as well as to dissect their dynamics *in vivo* [12].

In this review, we will focus our attention on *Coronavirinae*, a subfamily of RNA viruses consisting of four genera, namely *Alphacoronaviruses* (alpha-CoV), *Betacoronaviruses* (beta-CoV), *Gammacoronaviruses* (gamma-CoV), and *Deltacoronaviruses* (delta-CoV) [13]. Alpha-CoV and beta-CoV are able to infect humans, usually resulting in respiratory illness [14]. Beta-CoV comprise the three most pathogenic coronaviruses known to date: the severe acute respiratory syndrome virus (SARS-CoV), the Middle East respiratory syndrome virus (MERS-CoV), and the SARS-CoV-2 virus, responsible for the currently ongoing (December 2020) COVID-19 pandemic [15,16]. Coronaviruses (CoV) are characterized by the largest known positive-sense single-stranded RNA genomes (∼26–32 kb) [13], with a highly conserved architecture [17]. Despite the relatively poor fidelity of viral RNA-dependent RNA polymerases (RdRp), overall long-term integrity of such large genomes is ensured by the presence of the 3′–5′ exonuclease ExoN domain of the non-structural protein nsp14, that enables proof-reading at genome replication [18,19].

The genomic RNA (gRNA) has a 5′ cap and 3′ polyA tail, allowing direct translation of the non-structural polyproteins (nsp) ORF1a, and ORF1b, followed by assembly of the replicase-transcriptase complex (RTC). The RTC drives both genome replication and discontinuous transcription of subgenomic mRNAs (sgRNAs). Discontinuous transcription is mediated by short AU-rich transcription regulating sequences (TRSs), located either right downstream of the 5′ leader (TRS-L), or right upstream of each viral ORF (TRS-B), except for ORF1a and ORF1b [20]. The resulting sgRNAs are further translated to produce the structural proteins spike (S), envelope (E), membrane (M) and nucleocapsid (N), as well as several accessory proteins.

Over the past few months, enormous efforts have been put in trying to better understand the biology of the SARS-CoV-2 virus, to find an Achilles’ heel and to confine the COVID-19 pandemic. In a very short time, the structure and function of the SARS-CoV-2 proteome [21–25], transcriptome [26], as well as the interactions with the host cell proteome, both at the protein [27–29] and RNA levels [30,31], have been dissected. We will here summarize the current knowledge on coronavirus RNA structures and their regulation, as well as the most recent discoveries, as revealed by cutting-edge high-throughput analyses conducted on SARS-CoV-2.

## 5′ UTR structures

**Figure 1.**
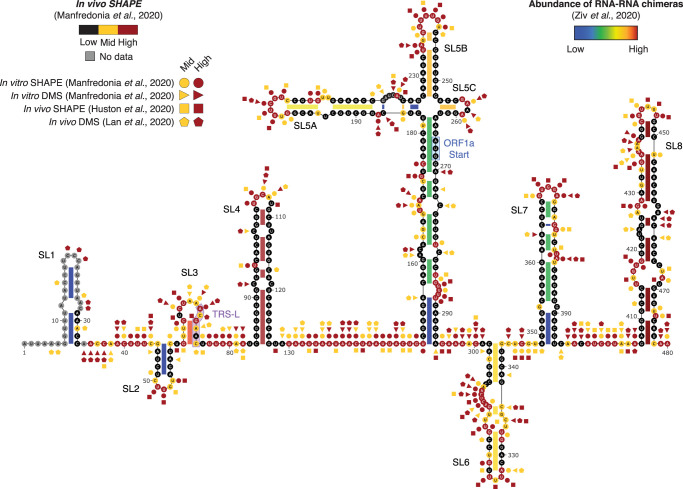
Structure of the SARS-CoV-2 5′ UTR. Secondary structure of the SARS-CoV-2 5′ UTR, with superimposed *in vivo* SHAPE reactivities from Manfredonia et al. [39]. Highly (red) and moderately (yellow) reactive residues from *in vitro* SHAPE (circles; Manfredonia et al. [39]), *in vitro* DMS (triangles; Manfredonia et al. [39]), *in vivo* SHAPE (squares; Huston et al. [36]) and *in vivo* DMS (pentagons; Lan et al. 2020) experiments are also indicated [36,37,39]. A higher reactivity indicates a higher propensity of bases to be single-stranded (for DMS), or structurally flexible (for SHAPE). Base-paired regions are color-coded according to the number of supporting chimeric reads from Ziv et al. [38]. The number of reads supporting the existence of SL8 was calculated by reanalyzing data from Ziv et al. [38] (GEO dataset: GSE154662).

The 5′ UTR of CoV is highly structured. A first consensus model of the 5′ UTR was originally proposed by comparative analysis of nine CoV from the three major CoV groups (alpha, beta, and gamma-CoV), and identified the three major conserved stem–loops SL1, SL2, and SL4 [32]. All three SLs have been consistently predicted and experimentally verified in SARS-CoV-2 [33–39]. Besides these, up to eight SLs can be present in CoV 5′ UTRs, with some degree of variation across different genera (Figure 1).

The SL1 has a functionally bipartite structure. On the one hand, the upper part of the SL1 must be folded. Indeed, destabilizing mutations introduced in the SL1 of Mouse hepatitis virus (MHV), a widely used model of beta-CoV, lead to defects in viral replication that can be rescued by compensatory mutations restoring the base-pairing [40]. On the other hand, the lower part of the SL1 needs to be structurally dynamic to establish a transient long-range interaction with the 3′ UTR, enabling the synthesis of subgenomic mRNAs (sgRNAs). Accordingly, studies conducted both in MHV and in human CoV 229E and NL63 (HCoV-229E and HCoV-NL63) showed that mutations in the lower part of the SL1 lead to defective sgRNA synthesis [41]. The SL1 appears to be highly functionally conserved. Indeed, replacement of the MHV SL1 with the SL1 of SARS-CoV has been proven to result in viable, yet more slowly replicating, chimeric viruses [42,43].

The SL2 is probably the most conserved cis-acting structure, as suggested by multiple phylogenetic analyses [32,42,44], hinting at its putative functional relevance. It consists of a highly conserved YUUGY pentaloop, stacked on a 5 bp-long helical stem, folding into a canonical CUYG tetraloop, with the 3′ pyrimidine flipping out of the stack [45]. Mutations disrupting the SL2 stem are not tolerated and lead to impaired sgRNA synthesis in MHV [32]. Accordingly, complete reversion of SL2-disrupted HCoV-229E mutants to the wild-type structure has been observed after just five passages of *in vitro* evolution [41]. Likewise, viability of mutants harboring mutations at G_4_ of the pentaloop is severely impaired, and spontaneous mutants of C_1_ > A were observed in G_4_ > U mutants, suggesting that these two bases are close enough in the loop to base-pair [46]. Mutants in which the SL2 of HCoV-229E has been replaced with the SL2 of either bovine CoV (BCoV) or SARS-CoV were vital and functional comparably to the wild-type virus, supporting the structural and functional conservation of the SL2 [41].

The SL3 (sometimes referred to as SL-II) is conserved only in a small subset of beta and gamma-CoV [44]. When present, this element encompasses the TRS-L. In MHV, although predicted, the existence of SL3 is not supported by *in vitro* Selective 2′ Hydroxyl Acylation analyzed by Primer Extension (SHAPE) analysis [47], with the TRS-L residing in a single-stranded region. In BCoV, HCoV-HKU1, and HCoV-OC43, the SL3 is predicted to form, with the TRS-L residing in the loop. Oppositely, in SARS-CoV and SARS-CoV-2 the TRS-L is predicted to reside in the 3′-half of the stem. This constitutes an exception, as in most CoV the TRS-L is predicted to be single-stranded [48], in agreement with its need to be available for interaction with the TRS-B sequences for proper sgRNA synthesis. Although both *in silico* prediction and *in vitro* SHAPE probing of the SARS-CoV-2 genome support folding of the SL3 [34,39], *in vivo* SHAPE and dimethyl sulfate (DMS) probing revealed intermediate reactivities in the stem of the SL3 [37,39], suggesting that this region might undergo dynamic unfolding to mediate genome cyclization. Direct capture of *in vivo* RNA–RNA interactions further supports this model, revealing the formation of a long-range interaction between the unfolded SL3 segment and the 3′ UTR [38].

The SL4 (sometimes referred to as SL-III) consists of a long bipartite hairpin (SL4a and SL4b, separated by an internal loop) located right downstream of the TRS-L, that appears to be conserved in all CoV genera [44]. This element harbors an upstream ORF (uORF) in over 75% of beta-CoV, encoding for a polypeptide of 3 to 13 amino acids. In MHV, mutations disrupting the coding potential of this uORF while preserving the structure of the SL4 resulted in the increased translation of ORF1ab, and were rapidly spontaneously reverted, although not impairing virus viability [49]. Complete deletion of the uORF start codon, however, resulted in defective viruses, and was spontaneously rescued by the appearance of a new uORF, suggesting that this uORF is involved in regulating the translation of ORF1ab. In MHV, the complete disruption of the SL4 structure, as well as the separate deletion of SL4a or SL4b, is tolerated, while the complete deletion of the SL4 is lethal [50]. These observations suggest that SL4 might function as a structural spacer, determining the proper orientation of the SL1, SL2, and TRS-L, possibly regulating the synthesis of sgRNAs. Recent *in silico* analysis of the SARS-CoV-2 genome suggested the presence of a second additional stem–loop, right downstream of the SL4 [34], whose existence, however, is supported neither by *in vitro* or *in vivo* structure probing analyses [37,39].

The SL5 partially encompasses the ORF1ab, including the initial portion of the *nsp1* gene, and is conserved in both alpha and beta-CoV [44]. It consists of a four-way junction, comprising three substructural stem–loops, SL5A, SL5B, and SL5C (sometimes referred to as SLIV, SLV, and SLVI). Folding of SL5 has been experimentally confirmed by *in vitro* probing of MHV [47], as well as by both *in vitro* and *in vivo* probing [36,37,39] and by direct *in vivo* RNA–RNA interaction capture in SARS-CoV-2 [38]. While the disruption of SL5A impairs viral replication in MHV, the importance of SL5C is more controversial [51]. Indeed, the deletion of SL5C in MHV is partially tolerated, but its disruption in BCoV viral defective interfering RNAs impairs replication [52]. However, Guan and colleagues pointed out that the mutants designed by Brown and collaborators could potentially affect the formation of the long-range SL5A base-pairing in BCoV, rather than affecting the folding of SL5C. To the best of our knowledge, no study has ever assessed the importance of SL5B. In alpha-CoV, each of the three hairpin loops presents the conserved sequence UUYCGU, while in beta-CoV this sequence is only present in the loops of SL5A and SL5B, and, in most cases (including SARS-CoV and SARS-CoV-2), the SL5C presents a GNRA tetraloop. MERS-CoV constitutes one of the few exceptions, as it harbors a non-conserved heptaloop. As viral encapsidation signals usually involve repeated structural elements, it has been previously proposed that the SL5 might act as a genome packaging signal in CoV [44], although this hypothesis has never been validated by reverse genetics approaches. CoV packaging signals will not be treated here, as they have already been extensively discussed in a recent review [53].

A number of additional SLs have been proposed downstream of the SL5. Although being located downstream of the start codon of ORF1ab, these additional structural elements are usually regarded as part of the 5′ UTR. SL6 and SL7 have been predicted computationally [54], and their existence is supported by *in vitro* probing of MHV [47], as well as by both *in vitro* and *in vivo* probing [33,36,37,39] and by direct *in vivo* RNA–RNA interaction capture in SARS-CoV-2 [38]. These two SLs appear to be less well conserved across beta-CoV [54], and targeted disruption of SL6 by mutagenesis in MHV does not affect viral replication nor viability [47]. Comparative computational analysis of the SARS-CoV-2 genome suggested the presence of three small additional SLs, approximately spanning nucleotides 398 to 450 [34]. However, existence of these SLs was not supported by *in silico* scanning of the SARS-CoV-2 genome in search for structure elements significantly more stable than expected by chance [55], nor by further *in vitro* and *in vivo* structure probing analyses conducted in the context of the full-length SARS-CoV-2 genome [37,39]. Rather, these analyses, as well as *in vivo* RNA–RNA interaction capture experiments [38], suggested the existence of a large SL8 element encompassing this region, spanning nucleotides 407 to 478.

## Ribosomal frameshifting element (FSE)

**Figure 2.**
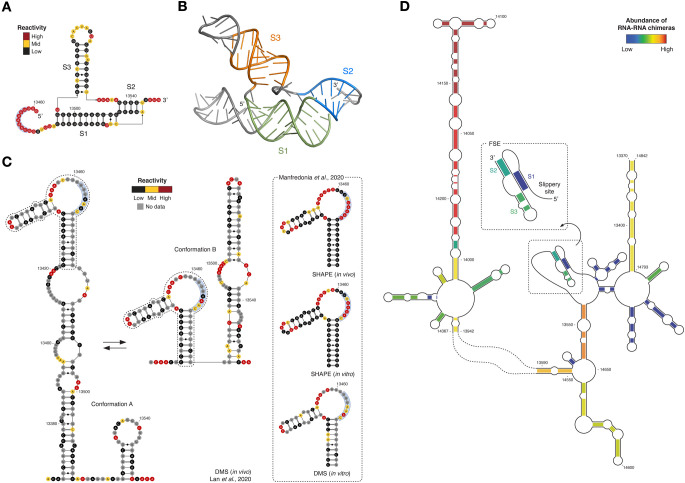
Structure models of the SARS-CoV-2 FSE. (**A**) Secondary structure of the SARS-CoV-2 FSE three-stem pseudoknotted conformation, with superimposed reactivities from Zhang et al. [59]. (**B**) Cryo-EM-derived structure of the SARS-CoV-2 FSE three-stem pseudoknotted conformation (PDB: 6XRZ). (**C**) On the left, the proposed secondary structure models of two coexisting mutually-exclusive alternative conformations of the SARS-CoV-2 FSE, as derived by *in vivo* DMS analysis, with superimposed reactivities from Lan et al. [37]. The alternative conformation of stem 1 is boxed. On the right, the same alternative conformation of stem 1 as confirmed by independent *in vitro* and *in vivo* DMS and SHAPE analyses, with reactivities superimposed from Manfredonia et al. [39]. The slippery site is boxed in blue. (**D**) Structure of the FSE-arch enclosing the FSE, as identified by direct RNA–RNA interaction mapping in the SARS-CoV-2 genome, with base-pairs colored according to their relative abundance from Ziv et al. [38].

Located at the intersection between ORF1a and ORF1b, the FSE is probably the most well studied structural element in CoV genomes. It regulates the programmed −1 ribosomal frameshifting, enabling the translation of ORF1b, partially overlapping ORF1a. Originally identified in the gamma-CoV infectious bronchitis virus (IBV), and further confirmed in MHV [56], it has been proposed to consist of a slippery UUUAAAC sequence, followed by a single-stranded spacer and a pseudoknotted stimulatory structure [57], composed of two stacked stems (S1 and S2), connected by two large loops (L1 and L2). In SARS-CoV [58] and in SARS-CoV-2 [34], the same overall organization has been suggested, but with a third stem–loop forming within L2 (S3). *In vitro* SHAPE and cryo-EM analyses of the isolated SARS-CoV-2 FSE confirmed this architecture (Figure 2A,B), additionally revealing two alternative tertiary arrangements, one of which shows the 5′ end threading through a ring formed within the pseudoknot, as opposed to an unthreaded conformation [59]. The threaded conformation might be responsible for the ribosomal frameshifting, via a torsional restraint mechanism. In agreement with the observed structure heterogeneity, molecular dynamics simulations of the SARS-CoV-2 FSE revealed multiple alternative packing of helices S1 and S2 [60]. Surprisingly, *in vitro* and *in vivo* DMS and SHAPE probing of SARS-CoV-2 suggest that the three-stem pseudoknot might not represent the predominant conformation in the context of the full genome (Figure 2C). Indeed, SHAPE analysis does not support the existence of helix S1 [39], and DMS-guided analysis of coexisting alternative conformations rather suggests the presence of two mutually-exclusive non-pseudoknotted stem–loop structures [37]. Accordingly, the abundance of chimeras from *in vivo* RNA–RNA interaction capture experiments supporting the existence of helix S1 appears to be extremely low [38] (Figure 2D). Furthermore, analysis of RNA–RNA interactions in both SARS-CoV-2 and MERS-CoV revealed that the FSE is embedded within a partially conserved ∼1.5 kb-long higher-order structure, bridging the 3′ end of ORF1a to the 5′ end of ORF1b, dubbed FSE-arch.

## 3′ UTR structures

Most of the knowledge on the 3′ UTR of coronaviruses comes from studies conducted on beta-CoV. This region appears to be structurally and functionally conserved within the same genus, so that the 3′ UTR of either BCoV or SARS-CoV can functionally replace that of MHV [61,62]. Oppositely, replacement of the MHV 3′ UTR with that of transmissible gastroenteritis virus (TGEV) or of IBV results in non-viable chimaeras, indicating that this functional equivalence is not consistent across different genera.

The most proximal structure element of the 3′ UTR, located immediately downstream of the stop codon of the nucleocapsid (N) gene, is the bulged stem–loop (BSL). This element shows very limited sequence, but high structural conservation across beta-CoV, and it has been proven to be essential for viral replication in MHV [62,63]. The terminal portion of the BSL, has been proposed to mediate the formation of an alternative, mutually-exclusive pseudoknotted conformation, by direct base-pairing to the loop of a hairpin (P2) located downstream of the BSL [64]. Originally identified in BCoV [65], it was proven to be essential for viral replication. The interconversion between the BSL and the pseudoknot structure has been proposed to act as a molecular switch, regulating viral RNA synthesis, and modulating the transition between different steps of the negative strand synthesis [66]. In alpha-CoV, although the BSL does not seem to be present, the pseudoknot is predicted to exist, and its formation appears to be mutually-exclusive with the presence of a small upstream stem–loop, suggesting that the putative molecular switch proposed in beta-CoV might also exist in alpha-CoV [48]. Thermal denaturation experiments conducted on the MHV 3′ UTR partially supported this model, revealing that the pseudoknot is highly dynamic: it only forms very weakly, and only when the terminal portion of the BSL cannot fold, suggesting that the fully folded BSL might represent the predominant conformation [67]. Accordingly, recent SHAPE-guided structure probing analyses, as well as direct capture of RNA–RNA interactions in the SARS-CoV-2 genome, did not support the presence of this pseudoknot [35,36,38,39]. It is however worth pointing out that the structural analyses performed in all these studies only presented an averaged snapshot of the SARS-CoV-2 RNA structure at a single time-point in a non-synchronous population of infected cells, while the existence of this pseudoknot might be temporally limited to a very specific stage of the viral life cycle.

The P2 stem–loop is part of a large multi-branched structure, known as the hypervariable region (HVR). The HVR shows poor sequence conservation across CoV, except for the octanucleotide GGAAGAGC, that appears to be extremely conserved across all CoV genera [48,68], although its functional relevance still needs to be assessed. It has been originally predicted and biochemically validated in MHV [69]. This region can tolerate extensive mutagenesis, and its complete deletion does not affect viral replication *in vitro* [68]. In contrast, removal of the HVR has a strong impact on viral pathogenicity, as demonstrated by the absence of any clinical sign of infection in mice infected with HVR-deleted viruses. Although the existence of this large structure in the SARS-CoV-2 genome is supported by both *in vitro* structure probing [39] and direct capture of *in vivo* RNA–RNA interactions [38], *in vivo* SHAPE probing reveals extensive unfolding of large part of the main HVR stem [36,39], suggesting that the HVR might adopt multiple alternative conformations *in vivo*.

Even if generally regarded as part of the 3′UTR, the BSL and part of HVR overlap a putative open reading frame (ORF10), whose existence, however, does not appear to be supported by a recent transcriptome analysis of SARS-CoV-2 infected cells [26].

A highly conserved structure element, stemming from the multi-branched loop of the HVR, is the stem–loop II-like motif (s2m). This stem–loop shows extreme sequence and structure similarity to the second stem–loop from the 3′ UTR of astroviruses and equine rhinovirus [70], and its existence in SARS-CoV-2 is supported under both *in vitro* and *in vivo* conditions by multiple studies [33,35–39]. X-ray crystallography of the s2m revealed that the tertiary folding of the apical pentanucleotide GAGUA loop resembles that of a typical GNRA tetraloop, with the U bulging out of the stack [71]. Overall, the s2m has a very particular geometry, forming a sharp 90° kink of the helix axis, resembling the 530 stem–loop of the *Escherichia coli* 16S ribosomal RNA, suggesting that this structure element might be involved in hijacking the host cell protein synthesis machinery.

## Other structural elements in the SARS-CoV-2 genome

**Figure 3.**
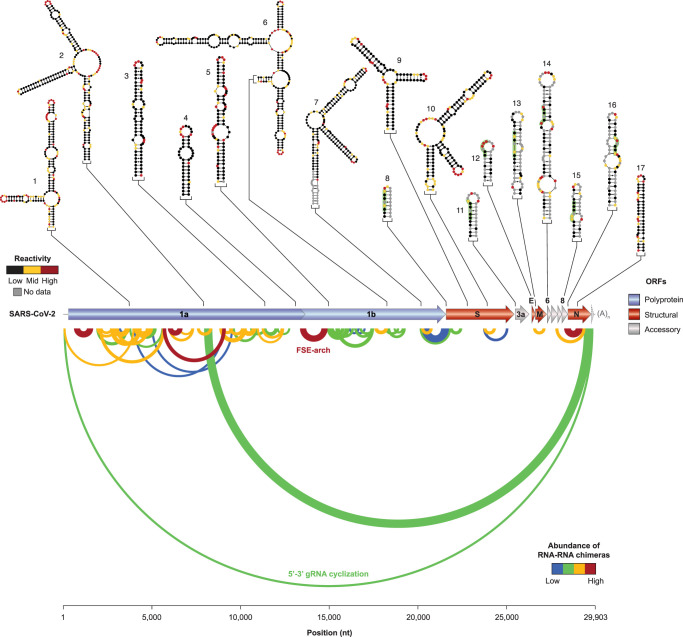
Landscape of SARS-CoV-2 RNA structures as revealed by high-throughput studies. On the top, the structures of conserved secondary structure elements, supported by significant covariation, as determined by SHAPE analyses of the SARS-CoV-2 genome, with superimposed reactivities from Manfredonia et al. [39] (1, 2, 6, 7, 9, 10, 17) and Huston et al. [36] (3, 4, 5). In addition, the structures of seven stem–loops (8, 11, 12, 13, 14, 15, 16) proposed to enclose the TRS-Bs (boxed in green), with superimposed reactivities from Lan et al. [37], are shown. On the bottom, an arc plot shows the long-range interactions identified by direct RNA–RNA interaction mapping in the SARS-CoV-2 genome, colored according to their relative abundance from Ziv et al. [38]. The FSE-arch is also indicated.

Besides the well-characterized structures within the 5′ and 3′ UTR, and the FSE, the SARS-CoV-2 genome is predicted to have an exceptionally high propensity to form stable RNA structures, significantly higher compared with that of other RNA viruses, including Hepatitis C virus (HCV), one of the most highly structured viral RNAs to date [72]. Indeed, recent genome-scale structure analyses of the SARS-CoV-2 genome, both *in vitro* and in living infected host cells, have led to the identification of a plethora of novel highly stable RNA structure elements, spread along the whole genome. DMS probing of infected host cells revealed that seven out of the nine TRS-Bs reside within stem–loop structures (Figure 3, top), characterized by different degrees of exposure of the TRS core sequence [37]. Indeed, while in four out of seven stem–loops (M, ORF6, ORF8, and N) the TRS core resides fully or partially within internal loops or single-nucleotide bulges, in three out of seven stem–loops (S, ORF3a, and E) the TRS core resides within base-paired helical regions. It is conceivable that these structures can regulate the accessibility of the TRS-B sequences, to finely tune the synthesis of the different sgRNAs. In line with this hypothesis, *in vivo* SHAPE analysis revealed a correlation between the degree of structuring of the TRS-B, and the relative abundance of the respective sgRNA [35]. Similarly, the RNA structural features were well correlated with the translation efficiency of sgRNAs, hinting at the importance of RNA structures in regulating the expression levels of viral proteins.

Comparison of the SARS-CoV-2 genome structure modeled by *in vivo* SHAPE analysis, with previously proposed structure models of Dengue (DENV) and HCV viruses, suggested that the SARS-CoV-2 genome is more prone to form locally-stable secondary structures, with fewer long-range base-pairing interactions, as compared with other positive-sense RNA viruses [36]. This preference for locally stable structures over long-range interactions might be involved in safeguarding the stability of SARS-CoV-2 genome and ensuring translation fidelity. Nonetheless, *in vivo* RNA–RNA interaction capture experiments revealed the existence of several long-range interactions, particularly enriched within ORF1a [38] (Figure 3, bottom). Part of these interactions appears to be mutually-exclusive, suggesting the presence of multiple coexisting alternative conformations. As an example, the 5′ UTR can interact with both the 3′ UTR, possibly to drive genome circularization, and with ORF1a.

Comparative *in vivo* and *in vitro* SHAPE probing analyses of the SARS-CoV-2 genome structure enabled the identification of nearly 87 well-defined structure elements, showing coherent folding under both conditions [39]. Of these, at least 10% showed significant covariation, indicating that they are under strong purifying selection, and appeared to be conserved, to different extents, in alpha-CoV, gamma-CoV, and delta-CoV as well (Figure 3, top). The same study revealed an unexpectedly high degree of correlation between the *in vivo* and *in vitro* conditions, suggesting that the sequence context and thermodynamics alone are major determinants in the folding of the SARS-CoV-2 genome. Accordingly, comparative DMS probing analyses of the FSE *in vivo* and *in vitro* showed that, *in vitro*, folding of the FSE region became progressively more and more similar to that observed *in vivo* when a larger portion of the surrounding sequence context was included in the *in vitro* transcript, with the DMS patterns being essentially indistinguishable when *in vitro* refolding the full SARS-CoV-2 genome [37].

Several studies have further started investigating the presence of RNA G-quadruplexes (G4s) in the SARS-CoV-2 genome [73–76]. Of the many predicted G4s, one G4 located within ORF1a (position 13385) was shown to bind and to be stabilized by BRACO-19 and TMPyP4, two known G4 binders, suggesting that these compounds might represent good scaffolds for the development of RNA-targeted small-molecule drugs [74]. Nevertheless, currently available studies only analyzed the candidate G4s under *in vitro* conditions. Further analyses will be needed to determine whether G4s can form in the SARS-CoV-2 genome, in the context of the infected cell.

## Additional insights from transcriptome-wide studies

Apart from intramolecular base-pairings, the SARS-CoV-2 genome appears to be involved also in a number of intermolecular RNA–RNA interactions with the host cell's transcriptome [38]. Particularly, direct capture of *in vivo* RNA–RNA interactions showed that the SARS-CoV-2 gRNA establishes several interactions with the small nuclear RNAs (snRNAs) U1, U2 and U4 at the level of ORF1ab, while the remainder of the gRNA is devoid of such interactions. Oppositely, the sgRNAs are enriched for interactions with the U1 and U2 snRNAs within the N gene and the 3′ UTR. Interactions with the U2 snRNA within ORF1a appear to be highly conserved, as confirmed by RNA–RNA interaction mapping in MERS-CoV-infected cells.

Additionally, gRNA pulldown experiments coupled to mass-spectrometry analyses showed that, during the SARS-CoV-2 life cycle, the gRNA establishes numerous RNA–protein interactions, both with viral and host cell proteins [30,31,77]. Among the top-enriched interactors is the SARS-CoV-2 N protein. Multiple computational and experimental analyses provided compelling evidences that the N protein has a high propensity to undergo liquid–liquid phase separation (LLPS), that is stimulated by interaction with the viral gRNA [78–81]. This interaction preferentially occurs with single-stranded RNA regions flanked by stably structured elements [82], further suggesting that LLPS might be exploited by SARS-CoV-2 for efficient genome packaging. Of the several interacting host cell proteins identified, the vast majority appears to have a host-protective effect, as their knockout resulted into virus-induced cell death [31], and to be part of the RNA–protein interactome of other positive-sense RNA viruses such as Zika virus (ZIKV) and DENV [30,31]. Of these, CNBP, a zinc-finger protein able to activate the expression of several innate immune response genes, was confirmed to directly bind the SARS-CoV-2 genome by enhanced cross-linking and immunoprecipitation (eCLIP) [30], suggesting that it might be involved in sensing foreign RNA. SHFL (also known as RyDEN), previously reported to inhibit the programmed -1 ribosomal frameshifting in human immunodeficiency virus (HIV) infections [83], was further shown to be able to inhibit ribosomal frameshifting by the SARS-CoV-2 FSE in a dual-color fluorescence reporter system. Interestingly, while the ZIKV and DENV genomes were found to be robustly associated with N6-methyladenosine (m^6^A) readers of the YTHDF family, and depleted of interactions with m^6^A erasers of the ALKBH family, the SARS-CoV-2 genome showed the opposite trend, suggesting that SARS-CoV-2 might escape m^6^A methylation to increase the gRNA stability [31]. Oppositely, a specific interaction with the mitochondrial 2′-O-methyltransferase MRM2 was observed, in agreement with the physical localization of the SARS-CoV-2 gRNA to mitochondria, as suggested by a recent machine learning analysis revealing the presence of several mitochondrial-localization signals in the 5′ UTR of the SARS-CoV-2 genome [84]. Direct RNA sequencing of the SARS-CoV-2 gRNA and sgRNAs further supports the notion that these RNAs might be post-transcriptionally modified [26,85]. Although the exact nature of these modifications is still unclear, a recent study suggests that at least 42 5-methylcytosine (m^5^C) sites might exist in SARS-CoV-2 RNAs [86].

## Concluding remarks

Recent advances in the RNA field have proven that RNA structure represents an ideal, yet largely underexploited, drug target [87,88]. In this perspective, the presence of a plethora of structured RNA elements in coronavirus genomes provides a unique opportunity for the development of new effective therapeutic strategies. Over the past few months, unprecedented efforts have been put in trying to dissect the complexity of the SARS-CoV-2 genome, revealing a multitude of previously unannotated RNA structure elements. Although their role has yet to be assessed, the conservation of a subset of these structures hints at their functional relevance. Importantly, preliminary analyses suggest that some of these elements might present druggable pockets [39], and early attempts to develop RNA-targeted therapeutic strategies are already underway [89].

## Perspectives

- Given the fast evolution of RNA viruses, leading to changes in the sequence and structure of viral proteins, the existence of conserved RNA structures in viral genomes provides a unique opportunity to develop more potent and durable antiviral therapeutic strategies
- Multiple groups have queried the structure of the SARS-CoV-2 genome under different conditions (*in vitro* or *in vivo*), using orthogonal probing approaches (DMS, NAI, NAI-N3), and reported diverse sets of RNA structures, providing a wide repertoire of potential therapeutic targets
- While most studies so far only provided a static snapshot of RNA structures in CoV genomes (at a single time-point of infection), future efforts should be aimed at characterizing the dynamics of the CoV RNA structurome to possibly identify crucial transient RNA folds and RNA structure switches, whose targeting might provide the means to effectively inhibit viral replication

## Open Access

Open access for this article was enabled by the participation of University of Groningen in an all-inclusive Read & Publish pilot with Portland Press and the Biochemical Society.

## Abbreviations

CoV: coronavirus
DMS: dimethyl sulfate
gRNA: genomic RNA
MERS: Middle-East respiratory syndrome
SARS: severe acute respiratory syndrome
sgRNA: subgenomic mRNA
SHAPE: Selective 2′ Hydroxyl Acylation analyzed by Primer Extension
